# The Treatment of Constitutional or True Syphilis

**Published:** 1875-03

**Authors:** Eugene Foster

**Affiliations:** Assistant Demonstrator of Anatomy, Medical College of Georgia, Augusta, Ga.


### THE TREATMENT OF CONSTITUTIONAL OR TRUE SYPHILIS.

*Extract from a Paper read before the Augusta Library and Medical Society.*

By EUGENE FOSTER, M.D.,
*Assistant Demonstrator of Anatomy, Medical College of Georgia, Augusta, Ga.*

It has become fashionable to talk of the natural history of syphilis. It is not uncommon to hear physicians assert that the failure to study the natural history of syphilis has caused us to attribute the recovery of the patient to medication, when really it was due to the fact that the disease ran its natural course, and terminated in recovery. Why, say they, do we resort to active medication in a disease which essentially tends to recovery? Why are we not the humble interpreters of nature's laws? Leave the disease to itself, and nature will eliminate the poison from the system. Active medication retards this process. Feed your patient upon full and nutritious diet, and give him the benefit of hygiene, and we have nothing to fear. Is this true science? Because nature unaided by art can perform this desirable task, does it necessarily follow that we may not advantageously avail ourselves of the aid of art? The accurate pictures and the masterly delineations of the natural history of this disease by syphilographers of undoubted authority teach us that the former conclusion was arrived at only through ignorance or prejudice, and that medication was resorted to only because of the disastrous consequences of inaction.

Leaving the question of the natural history of syphilis to those who possess a greater fondness for it than myself, I now come to the consideration of a subject which has distressed the medical mind ever since the discovery of the disease—the treatment of constitutional or true syphilis. The literature of the the subject is vast. If all that has been written upon this subject were collected into a single library, and it should be assigned to an energetic mind as a lifetime study to peruse in detail these works, none but the most daring would fail to become appalled at the magnitude of the task.

When I assert that mercury is the remedy above all others in the treatment of syphilis in all its stages, and that it is necessary to the cure of the disease, methinks I see that class of medical philosophers who boast of the high estate of anti-mercurialists hold up their hands in holy horror and exclaim, the rattling bones of the millions of human beings that have been immolated upon this altar rise up even now in ghostly array before their affrighted vision, and appeal to them in thunder tones, saying, permit me to return and warn my brother of the fate that is before him. There is no article in the materia medica that has met with such energetic opposition or unwarranted abuse as mercury. It has been denounced in the severest manner. It has been charged with producing secondary and tertiary syphilis, and woes and ills too numerous to mention. Its enemies, not satisfied with the power of invective, have resorted to the friendly aid of the picture gallery. Dr. Bennett holds up to our gaze the picture of the skeleton of a dog which has all the symptoms of tertiary syphilis in his bones, produced by mercury. This picture, with all its horror, is accompanied with the modest assertion that "the dog was frequently seen lapping vermilion oil paint, and that there can be no doubt but that large quantities of mercury were introduced into his system." A dog literally fed upon mercury; and, forsooth, the deduction is logical that if we feed a dog upon mercury, and the nutrition of the bones is destroyed, it must necessarily have the same effect upon his remote relative, man, if administered in the most minute doses! If mercury deserved one millionth part of the anathemas that have been pronounced against it, the profession should long since have expunged it from the materia medica, and petitioned the law-making power of the land to declare it a hanging offense for any man to have the audacity to prescribe it. Notwithstanding the hundreds of volumes that have been written against it in the most bitter manner, it stands out bolder than ever, and its claims are more firmly rooted and grounded in the love and veneration of thousands of medical philosophers, who to-day esteem it as the greatest boon left to fallen man in his suffering. To-day it stands where it stood more than two hundred years ago, universally, I had almost said, regarded as a specific for syphilis, and that it is necessary to the cure of that disease. Its perpetuity is the proof of its potency. Before entering in detail upon the treatment of the different stages of syphilis with this Samson of the materia medica, as it has been aptly called, let me premise that I cannot and will not submit to the assertion that I am obliged to refuse the aid of full and nutritious diet, the fulfillment of all the laws of hygiene and the aid of other medicines which increase the action of but cannot take the place of mercury. I claim that I am entitled to all these aids, and this can in no wise detract from the virtues of this agent.

If a patient comes to me with primary syphilis, I cauterize the chancre as soon as I decide the nature of it. Nitric acid is an excellent remedy for this purpose. The carbo-sulphuric paste is another excellent one. Lunar caustic, although much abused, comes well to the indication in these cases. Why should caustics be used? Does cauterization prevent constitutional infection? It does not, but if we thoroughly cauterize the chancre an eschar will fall about the seventh day, leaving a healthy granulating sore, which rapidly cicatrizes. I caution the patient to fulfill all the laws of hygiene and insist upon his taking nutritious food. If he is addicted to whisky drinking I inform him plainly that it is impossible to cure him until he gives up this terrible vice. I also proscribe the excessive use of tobacco. Induration being unmistakably an evidence that the system is already infected, I begin immediately the mercurial treatment.

If mercury be given in full doses in the incipiency of the chancre we will generally, or as a rule, prevent the supervention of secondary symptoms.

If we fail to prevent secondary symptoms we invariably ameliorate them.

Mark well the language of my first proposition. If mercury be given in the incipiency of the chancre—not when we first see it—we will generally prevent secondary syphilis.

But Dr. Bumstead meets us just here and asserts that my proposition is untrue, and says: "Although mercury retards the progress and probably ameliorates the secondary symptoms, yet it is a fact attested by many observers, myself included, that those cases ultimately do best in which specific treatment is deferred until the secondary stage." Brilliant idea! Why, sirs, the very language he uses appeals to the impartial mind to begin immediately the mercurial treatment. What does he, or I should say they, for there is a class of such medical philosophers, say is the advantage of such a course? They can't tell you why it is so, but experience proves that these cases ultimately do best in which mercury is deferred until the secondary symptoms appear. I deny this proposition. It is not founded upon either reason or experience. I affirm that we should begin mercurial treatment for the following reasons:

1st. Because this agent has the power of preventing secondary syphilis.

2d. That if we fail to prevent secondary symptoms we invariably ameliorate them.

Is our first proposition true? Listen to the voice of Ricord —the father of syphilography: "I say that if the treatment be well done and soon done—and this is most important—you can prevent the first bursting out of the secondary symptoms. Why this is not prevented is, that the treatment is applied too late in the first instance, and the secondaries often come on before the treatment of the primary is begun. But if you make the treatment of the primary early and effective, the secondaries will not appear; I can give you warrant for that."

Gentlemen, Ricord has touched the key-note of this whole affair. The reason why we have so many failures of mercury to prevent secondary syphilis is that that stage is already upon us before the treatment is begun. When a patient comes to us, from ten days to two weeks has generally elapsed from the time the chancre made its appearance. Then the physician, timid of this great agent, waits about an equal length of time to improve the stamina of his patient with tonics, etc., before beginning the specific treatment. Who that has any experience whatever in this disease will deny that secondary symptoms often appear in four weeks from the time the chancre was first discovered? I have seen this more than one time. Then, sirs, if we wait until the secondary stage is already upon us, is it wise or just to attribute the failure to the worthlessness of mercury in this stage, when in reality the fault is in the physician who has not used it properly? Listen to yet other testimony upon this point. Hutchinson says: "If mercury be given to a patient who has an undoubted indurated chancre, but in whom as yet no other constitutional symptoms are present, there is a fair amount of hope that it will prevent their occurrence." Each year of recent experience has made me increasingly convinced that this is the fact. I have used mercury each year more and more perseveringly, and begun it earlier; and the result has been that, whilst formerly, whenever the chancre was well indurated, I always expected constitutional symptoms in spite of treatment, I have come to regard their occurrence with any degree of severity as quite exceptional. I have treated many cases of the most typical chancres in which no constitutional symptoms whatever followed, although the patients remained under observation for long periods. In a still larger number, although some slight constitutional symptoms did appear, they were trivial in both character and duration. The sooner mercury is begun the less risk: the longer it is continued also the less risk. But why multiply testimony upon this point? Suffice it to say that Wallace of Dublin, Tournier, Verneil, Guerin, Hammond, Keys, Van Buren and many others of like eminence hold the same views. Who can be more capable of bearing testimony upon this point than the distinguished authors just quoted? Who can be more worthy of credence than they? What observation could be more philosophical? Upon what ground can their testimony be attacked? They are all men of large experience, astute observers, and philosophers in the strictest acceptation of the term. Men actuated by the dictates of wisdom, and who, unlike most authors, have studied the disease rather than books.

It is useless to consume your time in discussing my second proposition: *i. e.*, that if mercury fails to prevent secondary symptoms, it invariably ameliorates them. All authors, I had almost said, who deny that mercury has the power of preventing secondary syphilis, frankly admit that it does ameliorate them. This proposition, then, being admitted, I will not discuss it further than to say that, as it is impossible to tell to what extent the horrible ravages of this disease may go unless ameliorated, there is not a shadow of justice to the patient to allow it to remain in the system one moment longer than is necessary to drive it out by the most active medication.

As to the preparation of mercury which is most useful, also its mode of administration, I would say that this depends upon the circumstances of each individual case. No one preparation is alike useful in all cases. Just here permit me to remark that this agent properly used is potent for good—improperly used it is equally potent for harm. If I could do so I would write upon every bottle of this great medicine, in flaming capitals, "to use but not abuse." Great circumspection is demanded in the use of mercury. Its effects should be closely watched, and if it disagree with the patient we should change to another preparation, or if necessary withhold it for a while.

If we desire to bring the patient rapidly under the influence of mercury, and there are times when it is imperative to do so, calomel is in my opinion the best preparation. One grain taken daily will very soon have the desired effect. This amount may be given for several weeks, with freedom from salivation, if we administer fifteen grains of chlorate of potash with each dose of calomel. I have seen patients take this amount for three weeks, and no trace of salivation could be found. If the disease does not demand the speedy effect of the mercurial, I greatly prefer the bi-chloride to any other preparation. It has many advantages to commend it in practice. Prominently among its advantages I mention its freedom from salivation; it can be borne for months without detriment, in fact with marked benefit. It is also less apt to gripe or purge the patient than the other methods. I am in the habit of prescribing an eighth of a grain, with a teaspoonful of compound tincture of gentian, in a wineglass of water, three times daily. If the patient be quite cachectic it is judicious to administer the bi-chloride with the tincture of iron. If an eighth of a grain is not well borne, the dose should be reduced to a twelfth or sixteenth of a grain, and gradually arrive at an eighth. If it purge the patient, opiates should be combined with it. The protiodide is a favorite preparation, and in some cases valuable. It is more apt to produce intestinal irritation and abdominal pain than the bichloride. One grain, three times daily, is the usual amount administered. Opiates should always be combined with this preparation.

Inunction is a favorite method with many of the best syphilographers of the present as well as of the past. I am opposed to this method of introducing mercury into the system. It has many objections. The most serious one, and this is important, is that we cannot possibly know the amount of the medicine that reaches the system. It is also quite filthy and disgusting to the patient, and few of them can be induced to continue it for any length of time. It only offers advantage when we wish to introduce large quantities of mercury speedily into the system, and even then calomel is more advantageous.

Fumigation is another method of administering mercury. It is also troublesome and has no advantage over the other methods.

The hypodermic method has never been resorted to by me in practice, and therefore I can not speak of it from observation. In my opinion it offers no advantage over the internal method, and the evil consequences often following it more than counterbalance its good effects.

Whatever preparation of mercury we decide to use should be continued for a long time, never less than six months and better for twelve.

Mercury should not be used in patients affected with scrofula, tuberculosis or scurvy. Anæmia is also a contra-indication to its use, if it be not caused by syphilis. If anæmia is caused by syphilis then mercury is loudly called for and should be freely used. Unless we give mercury we can not cure the anæmia. Permit me to insist upon this point. I can testify from personal observation of its good effects in this condition. Do not waste your time in giving tonics to build up the blood preparatory to giving mercury. This is an error. No matter how energetically we use iron and other blood restoratives we fail to combat the anæmia until mercury is given. Be not timid of this great agent in these cases; use it freely but judiciously, and, my word for it, you will have the satisfaction of seeing the sanguine countenance of health succeed to pallor and weakness. I have given mercury freely to anæmic women, and have invariably had the satisfaction of seeing them become rosy in color and rapidly gain flesh. It is well to use the bichloride in the tincture of iron in these cases, though it is not necessary to have the iron in order to cure the anæmia; the mercury will do the work alone.

So far I have only spoken of the use of mercury in the primary stages of syphilis, excepting syphilitic anæmia, which applies to all stages of the disease. There is probably some excuse for those who do not use mercury in the primary stage of syphilis, but when we attempt to treat the secondary stage without this agent we are allowing this terrible disease to ravage the system of our patients when we can easily and successfully prevent it. There is therefore no excuse for the physician who is led astray by either the cry or prejudice. Candor compels me to say, that, with the accumulated experience of almost every one of the best syphilographers in all countries, they are culpable indeed who do not use mercury in secondary syphilis.

The treatment of tertiary syphilis has engaged the deepest study by men of all countries. The conclusions arrived at upon this point are various. I here reiterate what I have before stated, that mercury is the only agent which can effectually cure tertiary syphilis. Perhaps I am to be asked in what light do I regard the iodide of potassium? I reply that I regard it as an adjuvant to the treatment by mercury, *i.e.*, that it may increase the action of but cannot take the place of mercury. The effects of iodide of potassium upon tertiary symptoms are truly beautiful to witness, the symptoms disappearing under its use as though by magic; but the effect is exerted upon present symptoms only. No matter how thorough or prolonged the treatment by iodide of potassium may be, shortly after we discontinue its use the symptoms return. Resume the iodide, and when all symptoms have again disappeared, and we fancy that our patient has reached the haven of health, we are but listening to the voice of the syren, for very soon we will have the mortification of seeing tertiary symptoms reappear upon him. Bumstead, appreciating this lesson, has truly and forcibly said: "Unfortunately the iodides possess greater power to subdue tertiary symptoms for a time than to cause their permanent removal. The disease rapidly declines and disappears under their use, but in most cases returns in a few weeks or months after their suspension; and thus the patient becomes the slave of medicine or is obliged to resort to mercury for an effectual cure."

Lest you should think this a novel and untenable position, I will state that the same views are held by Sir Benjamin Brodie, Langston Parker and Mr. Hunt, of England, and Drs. Mussey, Willard Parker, John Watson, W. H. Van Buren, Blackman, W. H. Hammond, and many others of like eminence. Gentlemen, if there be one among us who has been startled upon hearing this proposition, let us consider it with unbiased minds, and if we be in error let us search diligently for the proper path and gladly pursue it. The exclusive use of iodide of potassium in tertiary syphilis is founded in error, and by reason of its many failures to cure the disease reflects discredit upon the profession of medicine. Its consequences upon the patient are also disastrous, for when he sees the disease return so often after he has thought himself cured, he will often fall into melancholia, which renders his life unpleasant, and thus syphilomania is often produced. The time allowed for reporting is too limited to permit me to theorize upon the mode of action of mercury, iodide of potassium, tartrate of iron and potassium, and other adjuvants in the treatment of syphilis. I do not know that, if the time would permit, I should be able to give you the mode of action of these drugs. We cannot rightfully be required to explain their mode of action. Enlightened experience should be the arbiter in these questions. Say what we will, enlightened experience is the very genius of medicine. Gentlemen, I have produced the testimony of many of the best syphilographers to the fact that iodide of potassium is not curative of tertiary syphilis. Mercury is the sheet-anchor of this as well as of the primary and secondary stages. If I have not demonstrated this proposition, then the authority of great names is worthless and large experience goes for nothing. If, then, I am required to throw aside this authority, I will continue to use mercury; for if I appeal to my own experience it tells me that it is the only agent which can effectually cure syphilis. It teaches me that when a patient comes to me who has taken pounds upon pounds of iodide of potassium, and whose treatment has extended over several years, and even then failed to be cured, that I can place him upon mercury and have the gratification of seeing the flush of health return to his cheeks and the tread of strength to his limbs. This is no picture drawn from fancy. It is the constant observation of hundreds of the most astute observers. I have seen patients who had taken the largest doses of iodide of potassium for months upon months and fail to obtain a cure, and then turning to mercury find that it yielded as though by magic. In some cases one week's course of mercury worked wonderful results.

I have not yet spoken of mercury as a specific for syphilis, but I will here say that if quinine is a specific for intermittent fever, or opium for pain, then mercury is doubly a specific for syphilis, for it will not fail half as often as the agents just named. One of the brightest omens for our science is to be found in the fact that the prejudice which has so long existed against mercury is rapidly vanishing away, and it is now being firmly established as the only agent which can thoroughly cure syphilis. So long as mercury can be found we are safe against the ravages of syphilis. Then let the waves of error and the storms of prejudice rise up against this grand old vessel; they will prove impotent to destroy her. She will spread her sails to the breeze and float triumphantly even in the thickest of the storm, override error and prejudice, and thus rightfully pinion upon her masthead, Mercury is the king of remedies in the treatment of syphilis.

NOTE.—I propose at a future time to give the notes of a number of cases of tertiary syphilis effectually cured by mercury after iodide of potassium had entirely failed; these cases being in my own practice.

